# *Photobacterium sanctipauli* sp. nov. isolated from bleached *Madracis decactis* (Scleractinia) in the St Peter & St Paul Archipelago, Mid-Atlantic Ridge, Brazil

**DOI:** 10.7717/peerj.427

**Published:** 2014-06-19

**Authors:** Ana Paula B. Moreira, Gwen Duytschaever, Luciane A. Chimetto Tonon, Adriana M. Fróes, Louisi S. de Oliveira, Gilberto M. Amado-Filho, Ronaldo B. Francini-Filho, Paul De Vos, Jean Swings, Cristiane C. Thompson, Fabiano L. Thompson

**Affiliations:** 1 Institute of Biology, Federal University of Rio de Janeiro (UFRJ), Rio de Janeiro, Brazil; 2 Botanical Garden Research Institute (JBRJ), Rio de Janeiro, Brazil; 3 Department of Environment and Engineering, Federal University of Paraíba (UFPB), Brazil; 4 BCCM/LMG Bacteria Collection, Ghent University, Ghent, Belgium; 5 Laboratory of Microbiology, Faculty of Sciences, Ghent University, Ghent, Belgium; 6 Laboratório de Sistemas Avançados de Gestão de Produção - SAGE - COPPE, Federal University of Rio de Janeiro, Rio de Janeiro, Brazil

**Keywords:** *Photobacterium sanctipauli*, St Paul’s rocks, Coral bleaching, New species, Genomic taxonomy

## Abstract

Five novel strains of *Photobacterium* (A-394T, A-373, A-379, A-397 and A-398) were isolated from bleached coral *Madracis decactis* (scleractinian) in the remote St Peter & St Archipelago (SPSPA), Mid-Atlantic Ridge, Brazil. Healthy *M. decactis* specimens were also surveyed, but no strains were related to them. The novel isolates formed a distinct lineage based on the 16S rRNA, *recA*, and *rpoA* gene sequences analysis. Their closest phylogenetic neighbours were *Photobacterium rosenbergii*, *P. gaetbulicola*, and *P. lutimaris*, sharing 96.6 to 95.8% 16S rRNA gene sequence similarity. The novel species can be differentiated from the closest neighbours by several phenotypic and chemotaxonomic markers. It grows at pH 11, produces tryptophane deaminase, presents the fatty acid C_18:0_, but lacks C_16:0_ iso. The whole cell protein profile, based in MALDI-TOF MS, distinguished the strains of the novel species among each other and from the closest neighbors. In addition, we are releasing the whole genome sequence of the type strain. The name *Photobacterium sanctipauli* sp. nov. is proposed for this taxon. The G + C content of the type strain A-394^T^ (= LMG27910^T^ = CAIM1892^T^) is 48.2 mol%.

## Introduction

Currently the genus *Photobacterium* comprises 26 formally described species (Euzéby, 2013; Liu et al., 2014; Srinivas et al., 2013). The habitats and isolation source include seawater (Reichelt, Baumann & Baumann, 1976; Yoshizawa et al., 2009), sea sediments (Jung et al., 2007; Seo et al., 2005a; Yoon et al., 2005), saline lake water (Rivas et al., 2006), and a variety of marine organisms with which the strains associate as commensals, saprophytes, bioluminescent symbionts, or pathogens (Urbanczyk, Ast & Dunlap, 2011). The list of hosts include fish (Liu et al., 2014; Onarheim et al., 1994; Ruimy et al., 1994), oyster and crab (Gomez-Gil et al., 2011), amphipods (Bartlett & Welch, 1995), sea hare (Seo et al., 2005b), squid (Kaeding et al., 2007) zoanthids (*Palythoa caribaeorum*) (Chimetto et al., 2010) and corals. *P. jeanii* and *P*. *rosenbergii* were the previously described species isolated from corals (Chimetto et al., 2010; Thompson et al., 2005b). *P. jeanii* was associated with healthy colonies of the scleractinian *Merulina ampliata* in Australia and the octocoral *Phyllogorgia dilatata* in Brazil (Chimetto et al., 2010), whereas *P*. *rosenbergii* was retrieved from several scleractinians, including healthy *Pachyseris speciosa* and diseased *M. ampliata, P. speciosa* and *Barabattoia amicorum*, in Australia (Thompson et al., 2005b), as well as from healthy *Mussismilia hispida* in Brazil (Chimetto et al., 2009). *Photobacterium* strains found in association with corals (healthy *Acropora palmata*) were identified as *P. phosphoreum*, *P. damselae* and *P. mandapamensis* (Ritchie, 2006). Coral microbiologists are challenged to increase our understanding in order to mitigate the worldwide spread of infectious diseases that are implicated in the decrease of coral cover in reef systems, markedly associated with climate changes and anthropogenic driven environmental disturbances (De’ath et al., 2012; Eakin et al., 2010; Mouchka, Hewson & Harvell, 2010; Rosenberg et al., 2007).

The study of the culturable heterotrophic microbiota of healthy and bleached *Madracis decactis* in the Brazilian St Peter & St Paul Archipelago (SPSPA) analyzed 403 isolates (Moreira et al., 2014). *P. angustum* and *P. damselae* were retrieved from healthy colonies, whilst five novel *Photobacterium* strains were only retrieved from the bleached corals. These five novel isolates originated from two colonies, but shared nearly identical 16S rRNA gene sequences. They showed less than 97% 16S rRNA gene sequence similarity towards the closest phylogenetic neighbor, *Photobacterium rosenbergii* (Moreira et al., 2014).

The present study aimed to describe a novel *Photobacterium* species, represented by five strains previously isolated in the SPSPA (Table S2), based on a polyphasic approach.

## Materials and Methods

All strains were isolated using thiosulfate-citrate-bile salt-sucrose (TCBS) medium at ambient temperature (∼27 °C) after 24–48 h incubation (Moreira et al., 2014). Gene sequences of 16S rRNA, recombination repair protein (*recA*), and RNA polymerase alpha subunit (*rpoA*) were obtained as described previously (Chimetto et al., 2009; Chimetto et al., 2008; Moreira et al., 2014). Primers used for gene amplification and sequencing were described in Moreira et al. (2014), Sawabe, Kita-Tsukamoto & Thompson (2007), Thompson et al. (2005a) and Thompson et al. (2001). Raw sequence data were transferred to ChromasPro V. 1.7.1 (Technelysium Pty. Ltd, Tewantin, Australia) where consensus sequences were determined. Pairwise similarities of these sequences with those of the closest phylogenetic neighbours were calculated using Jalview V.2 (Waterhouse et al., 2009). Sequences were aligned using ClustalW. Similarity matrices and phylogenetic analysis were performed by using the MEGA (Molecular Evolutionary Genetics Analysis) version 5.2 software (Tamura et al., 2011). Trees were drawn using the neighbour-joining method (Saitou & Nei, 1987). The robustness of each topology was checked by 1,000 bootstrap replications (Felsenstein, 1985). For genome sequencing 1ng of high quality DNA (obtained as in Moreira et al. (2014)) was used to construct the genomic paired-end library using the Nextera XT Sample Preparation Kit (Illumina^®^). Through this method, the DNA was simultaneously fragmented and tagged with sequencing adapters. The library size distribution was accessed using the 2100 Bioanalyzer and the High Sensitivity DNA Kit (Agilent^®^). The accurate quantification of the library was accomplished using the 7500 Real Time PCR (Applied Biosystems^®^) and the KAPA Library Quantification Kit (Kapabiosystems^®^). Paired-end (2 ×250 bp) sequencing was performed on a MiSeq (Illumina^®^) using the MiSeq reagent kit v2 (500 cycles). R1 and R2 reads were quality filtered (*Q* > 20) and 3’ end trimmed with Prinseq v0.20.4 (Schmieder & Edwards, 2011). Ray v. 2.3.1 was used to perform *De novo* assembly into scaffolds and contigs with default parameters (Boisvert et al., 2012). General genome features were determined through Rapid Annotations Using Subsystems Technology (The RAST server version 4.0) (Aziz et al., 2008). *In silico* DDH values were estimated to one strain of each *Photobacterium* species with publicly available genome using GGDC 2.0 (Auch, Klenk & Göker, 2010; Auch et al., 2010). This online tool infers genome-to-genome distances between pairs of entirely or partially sequenced genomes. Intergenomic distances are employed for wet-lab DDH prediction. Briefly, genome pairs were aligned with BLAST+ (Camacho et al., 2009) to generate a set of high-scoring segment pairs (HSPs). The information they contained (e.g., the total number of identical base pairs) was transformed into a distance value by the best-fit formula, according to (Meier-Kolthoff et al., 2013). DDH prediction from intergenomic distance, including confidence intervals, were provided by a tested generalized linear model (GLM, Nelder & Wedderburn, 1972) with log transformation (Meier-Kolthoff et al., 2013). Strains and genome accession numbers are in Table S1. AAI was calculated (according to Konstantinidis & Tiedje (2005)) towards the closest neighbor species determined by RAST (*P. leiognathi*). The gene sequence data obtained in this study are available through the open access website TAXVIBRIO (http://www.taxvibrio.lncc.br/). The GenBank accession numbers for the 16S rRNA, *recA*, and *rpoA* genes and genome sequences are listed in Table S1. The mol% G + C was determined according to Moreira, Pereira & Thompson (2011). MALDI-TOF MS protein profiles were determined as described previously (Wieme et al., 2012). Isolates were subcultured twice on MA for 24h at 30 °C. MALDI-TOF MS was conducted using a 4800 Plus MALDI-TOF/TOFTM Analyzer (Ab Sciex NV) in linear mode and the 4000 Series Explorer Software v3.5.3 (Applied Biosystems^®^). Spectra were generated with mMass software v5.5.0 (Strohalm et al., 2010). Type strains of the three closest related *Photobacterium* species were included for comparison. Phenotypic characterization was performed using commercial miniaturized kits (API 20E, API NE and API ZYM; BioMerieux) as described previously (Chimetto et al., 2010; Kim et al., 2010; Thompson et al., 2005b) and by BIOLOG GEN III metabolic fingerprinting (Biolog), following the manufacturer’s instructions. These tests included determination of temperature, pH and salinity growth ranges, several biochemical responses and 71 carbon source utilization assays. Unless indicated otherwise, isolates were grown onto MA for 24 hr at 30 °C. The optimal growth temperature was determined using TSB supplemented with 2.0% NaCl at pH 7.5, the optimal pH was determined in TSB supplemented with 2.0% NaCl at 30 °C and the optimal salinity was determined in peptone water (1.5% Peptone, 30 °C, pH 7.5). Growth under anaerobic conditions was determined after incubation in an anaerobic atmosphere (Microanaerobac, PROBAC, Brasil) on MA at 30 °C. Fatty acid methyl ester analyses were performed using the Sherlock Microbial Identification System (Royal Life Sciences Pvt. Ltd) according to the standard protocol. To this end, isolates were harvested from MA after 24 h of incubation at 30 °C. The results of these phenotypic analyses are presented in the species description and the distinctive features in Table 1.

**Table 1:** Phenotypic differences between *P. sanctipauli* sp. nov. and related *Photobacterium* species. Taxa: **1**, ***P. sanctipauli*** sp. nov. (five strains); **2**, ***P. rosenbergii*** LMG 22223^T^ (Srinivas et al., 2013; Thompson et al., 2005b); **3**, ***P. gaetbulicola*** Gung 47^T^ (Kim et al., 2010); **4**, ***P. lutimaris*** LMG 25278^T^ (Chimetto et al., 2010; Jung et al., 2007); **5**, ***P. jeanii*** LMG 25436^T^ (Chimetto et al., 2010; Srinivas et al., 2013); **6**, ***P. leiognathi*** LMG 4228^T^ (Baumann & Baumann, 1984; Chimetto et al., 2010; Nogi, Masui & Kato, 1998; Yoshizawa et al., 2009). +, Positive; −, negative; w, weak; v, variable; nd, no data available. All taxa are negative for Gram stain, lysine- and ornithine- decarboxylase, L-arabinose and D-sorbitol utilization; and positive for oxidase and alkaline phosphatase. Data in parentheses are for the type strains.

Characteristic	1	2	3	4	5	6
Salinity growth range (%)	1–8	1–7	0–8	1–6	0.5–4	0.5–6
Optimum NaCl concentration (%,w/v)	2–3	2–6	2–5	2–3	0.5–2	nd
Temperature growth range (°C)	15–42	15–35	10–40	4–41	15–37	nd-37
Optimum temperature (°C)	30	20–30	30	25–30	28	(26)
pH growth range	6–11	6–10	5–9	5–9	5–9	nd
Optimum pH	7.5	7–8.5	7–8	7.5–8.5	7–8	nd
**Enzyme activity**						
Catalase	w	(+)	+	w	+	(−)
Esterase (C4)	v(−)	+	+	+	+	+
Esterase lipase (C8)	v(−)	+	+	+	+	(+)
Lipase (C14)	−	(+ )	+	−	+	−
Leucine arylamidase	+	−	−	+	+	nd
Valine arylamidase	−	+	−	−	w	−
Cystine arylamidase	−	−	−	+	−	nd
Trypsin	−	−	−	−	+	(w)
Acid phosphatase	−	+	−	+	+	nd
Naphthol-AS-BI phosphohydrolase	+	+	−	+	+	+
*α*-galactosidase	−	(+)	−	−	−	−
*α*-glucosidase	−	(+)	−	−	+	(−)
N-acetyl-*β*-glucosaminidase	+	+	−	−	(−)	nd
*β*-galactosidase	+	+	−	−	+	+
Arginine dihydrolase	+	+	−	+	+	+
Tryptophane deaminase	v(w)	−	−	−	−	(−)
Indole production from tryptophan	v(−)	−	nd	+	−	(−)
Acetoin production from sodium pyruvate	−	−	nd	(−)	(w)	+
Gelatinase	−	−	nd	−	+	−
**Fermentation**						
Amygdalin	−	+	nd	(+)	−	(−)
Glucose	+	+	+	−	+	+
**Utilization as sole carbon source**						
Citrate	v(−)	+	+	+	−	−
D-Maltose	v(−)	(+)^*^	+	+	−	+
D-trehalose	v(−)	(+)^*^	+	+	−	−
D-Cellobiose	v(w)	(+)^*^	+	+	−	−
Sucrose	v(−)	(+ )^*^	+	+	v(+)	−
D-Raffinose	−	(−)^*^	+	+	nd	−
D-Melibiose	v(−)	(+)^*^	+	(−)	+	−
*β*-Methyl-D-Glucoside	v(−)	(+)^*^	nd	nd	nd	nd
D-Mannose	+	(+)^*^	+	+	−	+
D-Salicin	+	(+)^*^	−	+	nd	nd
D-Fructose	v(w)	(+)^*^	−	+	nd	−
L-Rhamnose	v(−)	(+ )^*^	−	−	−	−
D-Mannitol	v(−)	(+)^*^	+	−	−	−
*Myo*-Inositol	v(−)	(+)^*^	+	+	−	−
Tween 40	−	(w)^*^	+	+	nd	−
**DNA G + C content (mol%)**	48.2	47.6–47.9	50.6	48.3	49.8	41.6
**Fatty acids**						
C_16:0_ iso	−	1.9	0.4	−	1.9–3.5	−
C_18:0_	0.5–0.7	−	−	−	−	−

**Notes.**

* Data from this study.

## Results and Discussion

**Figure 1:**
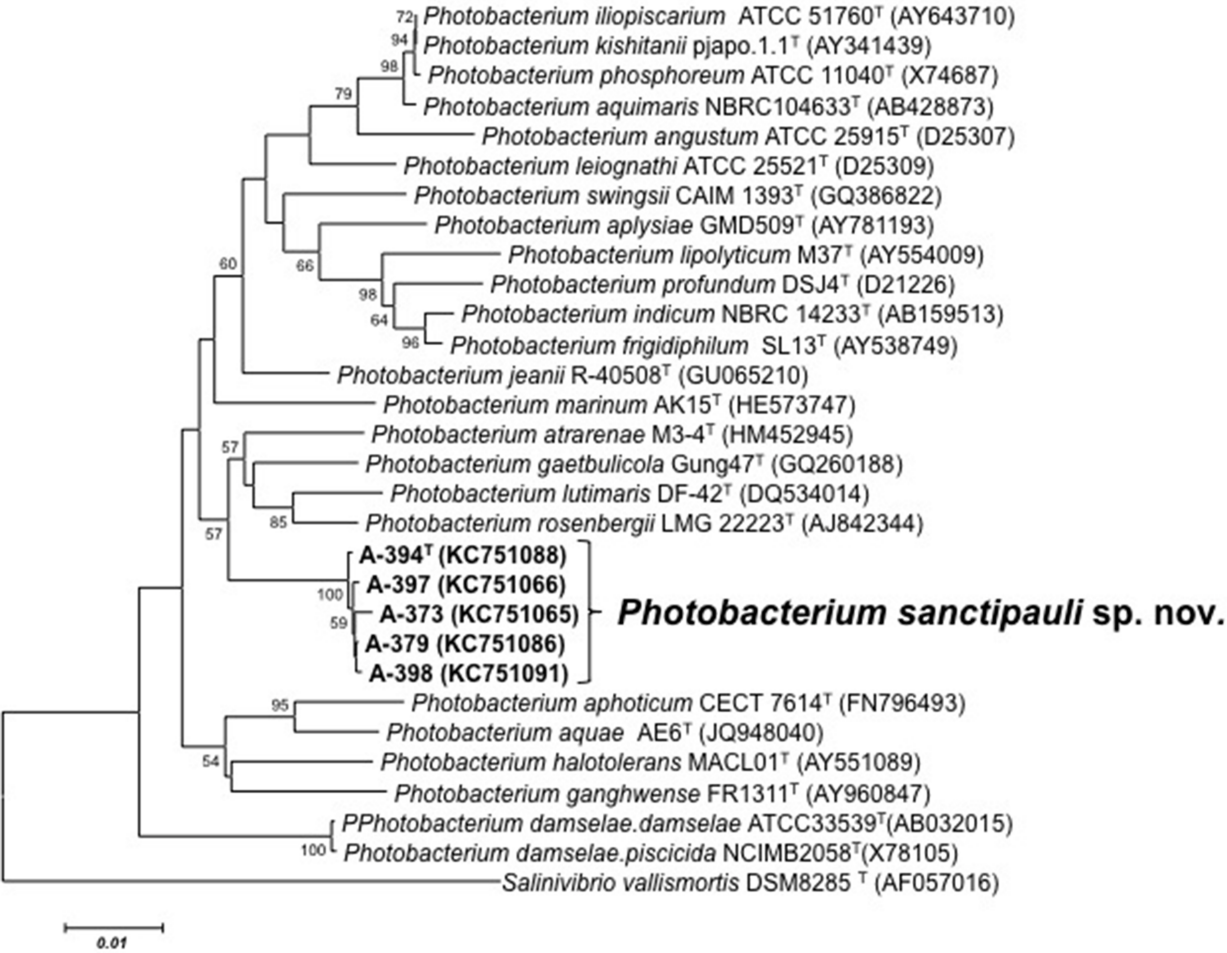
16S phylogenetic tree. Neighbour-joining phylogenetic tree of *Photobacterium* species based on 16S rRNA gene sequences (1,525 nt) showing the position of *P. sanctipauli* sp. nov. The optimal tree with the sum of branch length = 0.35538897 is shown. The evolutionary distances were computed using the Jukes-Cantor method. All positions containing alignment gaps and missing data were eliminated only in pairwise sequence comparisons (Pairwise deletion option). Phylogenetic analyses were conducted in MEGA5. Bootstrap values (>50%) based on 1,000 resamplings are shown. *Salinivibrio* was used as outgroup. Bar, 1% estimated sequence divergence.

**Figure 2:**
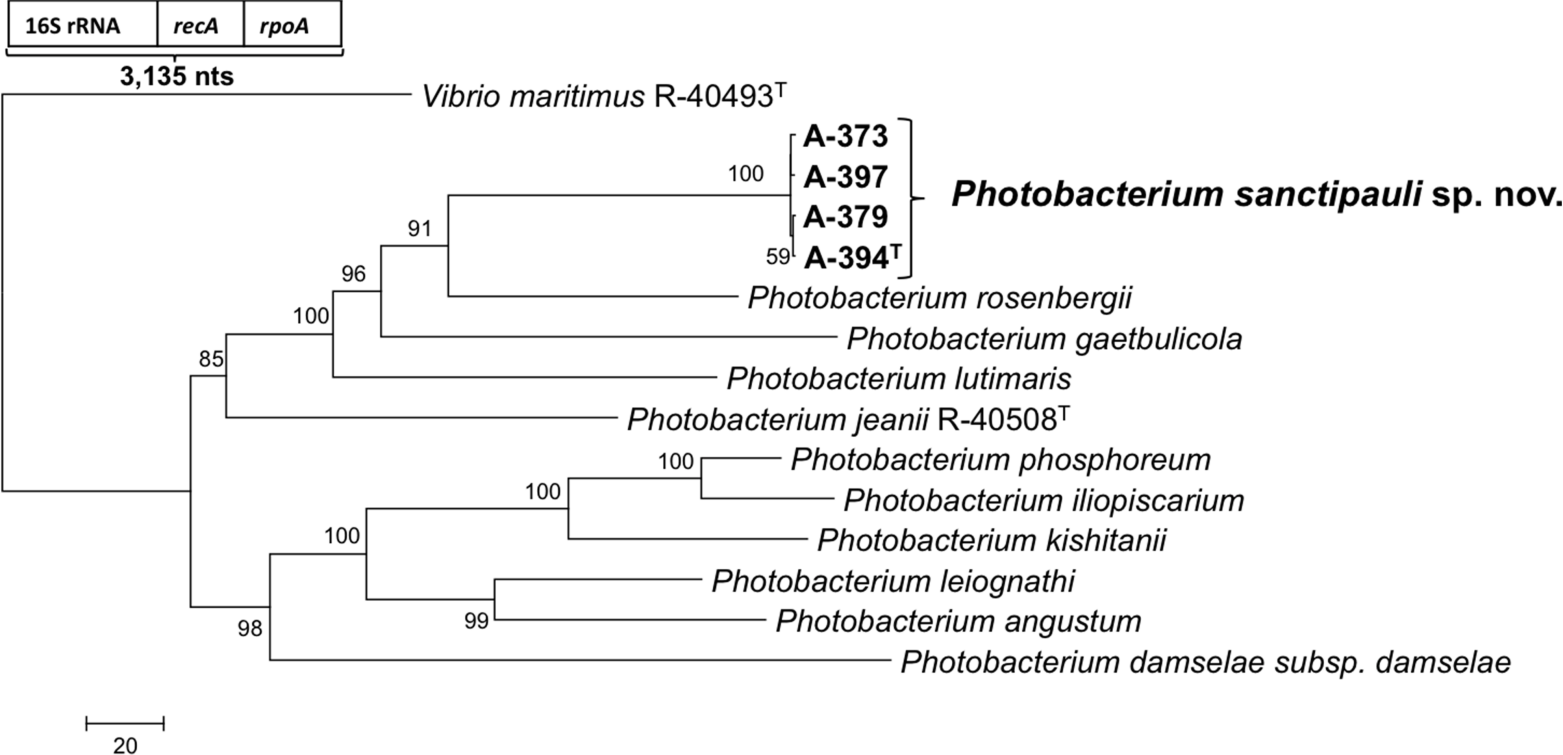
Neighbour-joining phylogenetic tree based on concatenated 16S rRNA, *recA* and *rpoA* gene sequences (3,135 nt) showing the position of *P. sanctipauli* sp. nov. The evolutionary distances were computed using the number of differences method and are in the units of the number of base differences per sequence. All positions containing alignment gaps and missing data were eliminated only in pairwise sequence comparisons (Pairwise deletion option). Phylogenetic analyses were conducted in MEGA5. Bootstrap values (>50%) based on 1,000 resamplings are shown. *Vibrio maritimus* R-40493^T^ was used as outgroup. Bar estimate sequence divergence.

16S rRNA gene sequence analysis revealed that the five isolates formed a tight monophyletic branch affiliated to the genus *Photobacterium* (Fig. 1). The five novel isolates shared more than 99% 16S rRNA gene sequence similarity. The sequence similarities towards the closest neighbours (based on 16S rRNA) were below the threshold (97%) established for species definition (Stackebrandt & Goebel, 1994; Vandamme et al., 1996). *P. rosenbergii* and *P. gaetbulicola* showed 96.6% sequence similarity, whereas *P. lutimaris* showed 95.8%. Other closely related neighbours have not been validly described yet. These are the cases of *P. atrarenae* (Kim et al., 2011) and *P. marinum* (Srinivas et al., 2013). The phylogenetic analysis based on 16S rRNA, *recA*, and *rpoA* gene sequences (3,135 nt in total) confirmed that the isolates formed a distinct lineage related to *P. rosenbergii* and *P. gaetbulicola* (Fig. 2). The novel isolates shared less than 87.2%, 96.5%, and 94.1% similarity based on *recA*, *rpo*A, and concatenated gene sequences (16S rRNA, *recA*, and *rpoA*) with their closest neighbours, respectively. These levels of similarity are below the cut-offs determined to define a species of the family *Vibrionaceae* (Thompson et al., 2009; Thompson et al., 2005a). The similarity levels between the novel isolates (A-394^T^, A-373, A-379, A-397 and A-398) ranged from 99.8% to 100% based on *recA*. Their *rpoA* sequences were identical. Trees based on partial sequences of the housekeeping genes *recA* (855 bp) and *rpoA* (969 bp) also confirmed their phylogenetic position in the genus *Photobacterium* and revealed they constituted a separate branch, clearly indicating that they belong to a new *Photobacterium* species (Figs. S1–S2). General features of A-394^T^ genome are supplied in Table S3. *In silico* DDH (%) values between A-394^T^ and *P*. *angustum* S14, *P. damselae* subsp. damselae CIP 102761, *P*. *halotolerans* DSM18316, *P. leiognathi* lrivu.4.1 and *P*. *profundum* 3TCK were 21.5 (±2.34), 22.7 (±2.37), 20.3 (±2.31), 21.6 (±2.35) and 20.6 (±2.31) respectively. AAI between A-394^T^ and *P. leiognathi* lrivu.4.1 CIP 102761 was 75%.

Several phenotypic features can be used to differentiate the novel species from its closest phylogenetic neighbors. The growth at pH 11, tryptophane deaminase activity, presence of the fatty acid C_18:0_, and absence of C_16:0_ iso (Table S4). MALDI-TOF MS protein profiles distinguished the novel strains among each other and from *P. rosenbergii* (LMG 22223^T^), *P. gaetbulicola* (LMG 27839^T^) and *P. lutimaris* (LMG 25278^T^) (Fig. S3). MLSA was more discriminative than MALDI-TOF and FAME for strain differentiation. Phenotypic and chemotaxonomic variation observed among the strains of the novel species indicate they are not clonal (Table S5 and Fig. S3). Based on the polyphasic analysis including MLSA, MALDI-TOF MS fingerprint profiles, chemotaxonomic and phenotypic tests presented in this study, we propose to classify the five isolates as a new species, *Photobacterium sanctipauli* sp. nov.

## Description of *Photobacterium sanctipauli* sp. nov.

*Photobacterium sanctipauli* (sanctí pauli N.L. gen. n. *sanctipauli* of Saint Paul, after the St Peter & St Paul Archipelago).

Colonies are small, beige, irregular shaped, with smooth and translucent edge and 1–2 mm in diameter after 24 h at 28 °C on MA under aerobic conditions. On TCBS colonies are green, round with a smooth border and 2–3 mm in diameter. Cells are small bacilli measuring 2–3 µm in diameter, Gram-negative, motile, facultative anaerobic, oxidase and catalase-positive. Grows well between 20 and 30 °C but not at 4 and 45 °C. No growth occurs in the absence of NaCl, but grows well under NaCl concentrations of 1%–8% (w/v). Grows at pH 6-11. Positive for alkaline phosphatase, leucine arylamidase, naphtol-AS-BI-phosphohydrolase, N-acetyl-*β*-glucosaminidase, *β*-galactosidase and arginine dihydrolase; but negative for lipase (C14), valine arylamidase, cystine arylamidase, trypsin, *α*-chemotrypsin, acid phosphatase, *α*-galactosidase, *β*-glucuronidase, *α*-glucosidase, *β*-glucosidase, *α*-mannosidase, *α*-fucosidase, lysine decarboxylase, ornithine decarboxylase, H_2_S production, urease activity, acetoin production (Voges–Proskauer) and gelatinase. Variable reactions were obtained for esterase (C4) (−), esterase lipase (C8) (−), tryptophane deaminase (w) and indole production (−) (whenever variable within species, result for the type strain is in parentheses). Reduces nitrate to nitrite but not to N_2_. Positive for fermentation/oxidation of glucose and mannitol but negative for inositol, sorbitol, rhamnose, saccharose, amygdalin and arabinose. Melibiose (+) gave variable reactions. D-Salicin, *α*-D-glucose, D-mannose, D-galactose are used as sole energy sources. Does not utilize dextrin, D-raffinose, glycerol, N-acetyl-D-galactosamine, D-glucose-6-PO4, D-aspartic acid, D-serine, gelatin, glycyl-L-proline, L-alanine, L-arginine, L-aspartic acid, L-glutamic acid, L-pyroglutamic acid, L-serine, pectin, L-galactonic acid lactone, mucic acid, quinic acid, D-saccharic acid, p-hydroxy-phenylacetic acid, methyl pyruvate, D-lactic acid methyl ester, citric acid, D-malic acid, bromo-succinic acid, *γ*-amino-butyric acid, *α*-hydroxy-butyric acid, *β*-hydroxy-D,L-butyric acid, propionic acid, acetic acid and formic acid. The following reactions are variable within the species: citrate (−), D-maltose (−), D-trehalose (−), D-cellobiose (w), gentiobiose (−), sucrose (−), D-turanose (−), stachyose (−), *α*-D-lactose (−), D-melibiose (−), *β*-methyl-D-glucoside (−), N-acetyl-D-glucosamine (−), N-acetyl-*β*-mannosamine (−), N-acetyl neuraminic acid (−), D-fructose (−), 3-methyl glucose (w), D-fucose (w), L-fucose (w), L-rhamnose (−), inosine (−), D-sorbitol (−), D-mannitol (−), D-arabitol (−), myo-inositol (−), D-glucose-6-PO4 (−), L-histidine (w), D-galacturonic acid (−), D-gluconic acid (−), D-glucuronic acid (−), glucuronamide (w), L-lactic acid (−), *α*-keto-glutaric acid (w), L-malic acid (−), tween 40 (−) and acetoacetic acid (w). Does not assimilate any of the substrates included in the API 20 NE system. The most abundant cellular fatty acids are summed feature 3 (43.5%; comprising C_16:1_*ω*7*c* and/or iso-C_15_ 2-OH), C_16:0_ (21.4%), C_18:1_*ω*7*c* (11.6%), C_14:0_ (5.2%), C_12:0_ and summed feature 2 (3.7%; comprising C_12:0_ ALDE, iso-C_16:1_I and/or C_14:0_ 3–OH and/or an unidentified fatty acid with equivalent chain length of 10.928), C_12:0_3–OH(2.5%), C_17:0_ (1.6%), Iso-C_17:0_ (1.5%), Iso-C_15:0_ and C_17:1_*ω*8*c* (1.1%), and in minor amounts C_13:0_, C_17:1_*ω*6*c*, C_18:0_ and Unknown 12.484 (0.3–0.5%). The G + C content of the type strain (A-394^T^) is 48.2 mol%. The type strain is A-394^T^ (= LMG 27910^T^ = CAIM 1892^T^). It was isolated from the tissues of bleached *Madracis decactis* (Scleractinia) in St Peter & St Paul Archipelago, Brazil.

**Abbreviations**
SPSPA: St Peter & St Paul Archipelago
MLSA: multilocus sequence analysis
AAI: average amino acid identity
DDH: DNA-DNA hybridization
GGDC: Genome-To-Genome Distance Calculator
FAME: fatty acid methyl ester analyses
MALDI-TOF: matrix-assisted laser desorption/ionization time-of-flight

## Supplemental Information

10.7717/peerj.427/supp-1 Table S1 GenBank accession numbers for genes and genomes **Upper** GenBank accession numbers for the 16S rRNA gene, *recA* and *rpoA* housekeeping genes and genome sequences of *Photobacterium sanctipauli* sp. nov.; and for *recA* and *rpoA* of *P. gaetbulicola* LMG 27839^T^ (data from this study). **Lower** Accession numbers for the *Photobacterium* strains’ genomes used for GGD calculation (data publicly available at GenBank).

10.7717/peerj.427/supp-2 Table S2 Strains and source information Strains of *Photobacterium sanctipauli* sp. nov. and source information.

10.7717/peerj.427/supp-3 Table S3 Statistics and general features of the type strain’s genome Statistics and general features of A-394^T^ (= LMG 27910^T^ = CAIM1892^T^) genome determined in RAST environment.

10.7717/peerj.427/supp-4 Table S4 Cellular fatty acids content of *Photobacterium sanctipauli* sp. nov. and related taxa of the genus *Photobacterium* Taxa: **1**, *P. sanctipauli* (range profile of five strains); **2**, *P. rosenbergii* LMG 22223^T^ (Thompson et al., 2005b); **3**, *P. gaetbulicola* Gung 47^T^; and **4**, *P. lutimaris* LMG 25278^T^ (Kim et al., 2010). Summed feature 2 comprises C_12:0_ ALDE, iso-C_16:1_ I and/or C_14:0_ 3-OH and/or an unidentified fatty acid with equivalent chain length of 10.928. Summed feature 3 comprises C_16:1_*ω*7*c* and/or iso-C_15_ 2-OH. Data are expressed as percentages of total fatty acids. Fatty acids representing <1% are not shown, except for C_18:0_, because it was represented in all strains with approximately the same intensity.

10.7717/peerj.427/supp-5 Table S5 Phenotypic variability amongst strains of *P. sanctipauli* sp. nov Phenotypic variability amongst representative strains of *P. sanctipauli* sp. nov. +, positive; −, negative; w, weak

10.7717/peerj.427/supp-6 Figure S1 *recA phylogenetic tree*
Neighbour-joining phylogenetic tree showing the position of *P. sanctipauli* sp. nov based on *recA* gene sequences (855 bp). The evolutionary distances were computed using the Kimura 2-parameter method. Phylogenetic analyses were conducted in MEGA5. Bootstrap values (>50%) shown are based on 1,000 repetitions. *Vibrio maritimus* R-40493^T^ was used as outgroup. Bar, 2% estimated sequence divergence.

10.7717/peerj.427/supp-7 Figure S2 *rpoA* phylogenetic tree Neighbour-joining phylogenetic tree showing the position of *P. sanctipauli* sp. nov based on *rpoA* gene sequences (969 bp). The evolutionary distances were computed using the Kimura 2-parameter method. Phylogenetic analyses were conducted in MEGA5. Bootstrap values (>50%) shown are based on 1,000 repetitions. *Vibrio maritimus* R-40493^T^ was used as outgroup. Bar, 1% estimated sequence divergence.

10.7717/peerj.427/supp-8 Figure S3 MALDI-TOF MS fingerprint profiles of the 5 novel strains and the closely related type strains Comparison of the MALDI-TOF MS fingerprint profiles of the 5 novel strains (**A**−**394^T^**, A-373, A-379, A-397 and A-398) showing they are not clonal. The closely related type strains of *P. rosenbergii* (LMG 22223^T^), *P. gaetbulicola* (LMG 27839^T^) and *P. lutimaris* (LMG 25278^T^) were included in the analysis. The dendrogram was constructed using Pearson correlation coefficient and UPGMA.

## Additional Information and Declarations

### Field Study Permissions

The following information was supplied relating to field study approvals (i.e., approving body and any reference numbers): Sampling permit Sisbio no. 24732-1 issued by the Ministry of Environment Institute Chico Mendes (ICMBio).

### DNA Deposition

The following information was supplied regarding the deposition of DNA sequences: Nucleotide sequence data for Photobacterium sanctipauli sp. nov are available in the DDBJ/EMBL/GenBank databases under the following accession number(s): KC751065-6, KC751086, KC751088, KC751090-1 (16S rRNA); KF748538-41 (recA), KF748542-5 (rpoA) and ASHX00000000 for whole genome sequence. *P. gaetbulicola* LMG 27839T reported nucleotide sequence accession numbers: KF771650 (recA) and KF771651 (rpoA). P.sanctipauli: This Whole Genome Shotgun project has been deposited at DDBJ/EMBL/GenBank under the accession JGVO00000000. The version described in this paper is version JGVO01000000.

### Data Deposition

The following information was supplied regarding the deposition of related data: Gene sequences are deposited at http://www.taxvibrio.lncc.br/, Genbank.

### New Species Registration

The following information was supplied regarding the registration of a newly described species: The type strain A-394^T^ have been deposited in two culture collections: BCCM/LMG Bacteria Collection (Belgium) = LMG 27910^T^, and Collection of Aquatic Important Microorganism CAIM (Mexico) = CAIM 1892^T^. Cultures are also deposited in the brazilian collection Coleção de Bactérias do Ambiente e Saúde http://cbas.fiocruz.br/ for open access.
