# Supplementary material for: Photobacterium sanctipauli sp. nov. isolated from bleached Madracis decactis (Scleractinia) in the St Peter & St Paul Archipelago, Mid-Atlantic Ridge, Brazil
Source: PeerJ. 2014 Jun 19;2:e427. doi: 10.7717/peerj.427 (PMC4081156; doi:10.7717/peerj.427)
Supplement: Table S1 — Upper GenBank accession numbers for the 16S rRNA gene, recA and rpoA housekeeping genes and genome sequences of Photobacterium sanctipauli sp. nov.; and for recA and rpoA of P. gaetbulicola LMG 27839T (data from this study). Lower Accession numbers for the Photobacterium strains’ genomes used for GGD calculation (data publicly available at GenBank). [file peerj-02-427-s001.pdf]

Table S1. **Upper** GenBank accession numbers for the 16S rRNA gene, *recA* and *rpoA* housekeeping genes and genome sequences of *Photobacterium sanctipauli* sp. nov.; and for *recA* and *rpoA* of *P. gaetbulicola* LMG 27839^T (data from this study). **Lower** Accession numbers for the *Photobacterium* strains' genomes used for GGD calculation (data publicly available at GenBank).

Strains	16S rRNA	*recA*	*rpoA*	Genomes
**A-394^T**	KC751088	KF748540	KF748544	JGVO00000000
**A-373**	KC751065	KF748538	KF748542	
**A-379**	KC751086	KF748539	KF748543	
**A-397**	KC751086	KF748541	KF748545	
**A-398**	KC751091			
P. gaetbulicola LMG 27839^T		KF771650	KF771651	
P. angustum S14				AAOJ00000000.1
P. damselae subsp. damselae CIP 102761				ADBS00000000.1
P. halotolerans DSM18316				AULG00000000.1
P. leiognathi lrivu.4.1				BANQ00000000.1
P. profundum 3TCK				AAPH00000000.1
