# Supplementary material for: Photobacterium sanctipauli sp. nov. isolated from bleached Madracis decactis (Scleractinia) in the St Peter & St Paul Archipelago, Mid-Atlantic Ridge, Brazil
Source: PeerJ. 2014 Jun 19;2:e427. doi: 10.7717/peerj.427 (PMC4081156; doi:10.7717/peerj.427)
Supplement: Table S2 — Strains of Photobacterium sanctipauli sp. nov. and source information. [file peerj-02-427-s002.pdf]

Table S2. Strains of *Photobacterium sanctipauli* sp. nov. and source information

Isolate	Source	Location<br>Isolation date	Reference
A-373 (CAIM 1893)<br>A-379 (R-52166)<br>**A-394^{T}**<br>**(LMG 27910^{T}; CAIM 1892^{T})**<br>A-397 (R-52168)<br>A-398 (R-52169)	Bleached tissues<br>*Madracis decactis*<br>(Scleractinia)	St Peter & St Paul<br>Archipelago<br>Mid-Atlantic Ridge<br>Brazil<br>00°56’N; 29°22’W<br>22/nov/2010	(Moreira et al. 2014)

Reference

Moreira APB, Tonon LAC, Cecilia do Valle PP, Alves Jr N, Amado-Filho GM, Francini-Filho RB, Paranhos R, and Thompson FL. 2014. Culturable Heterotrophic Bacteria Associated with Healthy and Bleached Scleractinian *Madracis decactis* and the Fireworm *Hermodice carunculata* from the Remote St. Peter and St. Paul Archipelago, Brazil. *Curr Microbiol* 68:38-46.
