# Supplementary material for: Photobacterium sanctipauli sp. nov. isolated from bleached Madracis decactis (Scleractinia) in the St Peter & St Paul Archipelago, Mid-Atlantic Ridge, Brazil
Source: PeerJ. 2014 Jun 19;2:e427. doi: 10.7717/peerj.427 (PMC4081156; doi:10.7717/peerj.427)
Supplement: Table S3 — Statistics and general features of A-394T (= LMG 27910T = CAIM1892T) genome determined in RAST environment. [file peerj-02-427-s003.pdf]

Table S3. Statistics and general features of A-394^{T} (=LMG 27910^{T}=CAIM 1892^{T}) genome determined in RAST environment:

**Features**	**as uploaded**	**after splitting into scaffolds**
Size (bp)	5,725,370	5,718,047
Contigs	1,805	1,830
Shortest contig size	100	100
Median contig size	2,005	1,988
Mean sequence size	3,172.0	3,124.2
Longest contig size	27,307	24,391
Content GC (%)	48.1	48.1
**Subsystems (n=499)**		
RNAs (t,rRNAs)	58	
Predicted ORFs	5,008	
Features (%)	In Subsystem=47	Not in subsystem=53
Total ($n^{\underline{0}}$)	2,353	2,655
Hypothetical Proteins ($n^{\underline{0}}$)	144	1,301
Non-Hypothetical ($n^{\underline{0}}$)	2,209	1,354
