# Supplementary material for: Photobacterium sanctipauli sp. nov. isolated from bleached Madracis decactis (Scleractinia) in the St Peter & St Paul Archipelago, Mid-Atlantic Ridge, Brazil
Source: PeerJ. 2014 Jun 19;2:e427. doi: 10.7717/peerj.427 (PMC4081156; doi:10.7717/peerj.427)
Supplement: Table S4 — Taxa: 1, P. sanctipauli (range profile of five strains); 2, P. rosenbergii LMG 22223T (Thompson et al., 2005b); 3, P. gaetbulicola Gung 47T; and 4, P. lutimaris LMG 25278T (Kim et al., 2010). Summed feature 2 comprises C12:0 ALDE, iso-C16:1 I and/or C14:0 3-OH and/or an unidentified fatty acid with equivalent chain length of 10.928. Summed feature 3 comprises C16:1ω7c and/or iso-C15 2-OH. Data are expressed as percentages of total fatty acids. Fatty acids representing <1% are not shown, except for C18:0, because it was represented in all strains with approximately the same intensity. [file peerj-02-427-s004.pdf]

Table S4. Cellular fatty acids content of *Photobacterium sanctipauli* sp. nov. and related taxa of the genus *Photobacterium*. Taxa: **1**, *P. sanctipauli* (range profile of five strains); **2**, *P. rosenbergii* LMG 22223^T (Thompson et al. 2005); **3**, *P. gaetbulicola* Gung47^T; and **4**, *P. lutimaris* LMG 25278^T (Kim et al. 2010). Summed feature 2 comprises $C_{12:0}$ ALDE, iso-$C_{16:1}$ I and/or $C_{14:0}$ 3-OH and/or an unidentified fatty acid with equivalent chain length of 10.928. Summed feature 3 comprises $C_{16:1}\omega 7c$ and/or iso-C_{15} 2-OH. Data are expressed as percentages of total fatty acids. Fatty acids representing <1 % are not shown, except for $C_{18:0}$, because it was represented in all strains with approximately the same intensity

**Fatty acids**	**1**	**2**	**3**	**4**
$C_{12:0}$	3.7–4.5	2	1.7	2.4
$C_{12:0}$ 3-OH	2.5-3.6	2-3	2.8	2.6
$C_{14:0}$	4.7-5.8	3-4	4.2	2.4
$C_{15:0}$ iso	0.6-1.1	2-4	6.9	5
$C_{16:0}$ iso	-	1.9	0.4	-
$C_{16:0}$	18.5-21.4	10-15	14	13.1
$C_{17:0}$	1.2-1.6	1	1	-
$C_{17:0}$ iso	0.7-1.5	3-6	3.5	1.8
$C_{17:1\omega 8c}$	0.3-1.1	1	0.6	-
$C_{18:0}$	0.5-0.7	-	-	-
$C_{18:1\omega 6c}$	0-3.3	-	3.1	-
$C_{18:1\omega 7c}$	7.9-12.7	17-19	10.3	13
Summed feature 2	3.7-4.7	2-3	3.7	3
Summed feature 3	43-45.5	41-44	33.6	43.3

References

Kim Y-O, Kim K-K, Park S, Kang S-J, Lee J-H, Lee S-J, Oh T-K, and Yoon J-H. 2010. Photobacterium gaetbulicola sp. nov., a lipolytic bacterium isolated from a tidal flat sediment. *Int J Syst Evol Microbiol* 60:2587-2591.

Thompson F, Thompson C, Naser S, Hoste B, Vandemeulebroecke K, Munn C, Bourne D, and Swings J. 2005. Photobacterium rosenbergii sp. nov. and Enterovibrio coralii sp. nov., vibrios associated with coral bleaching. *Int J Syst Evol Microbiol* 55:913-917.
