# Supplementary material for: Photobacterium sanctipauli sp. nov. isolated from bleached Madracis decactis (Scleractinia) in the St Peter & St Paul Archipelago, Mid-Atlantic Ridge, Brazil
Source: PeerJ. 2014 Jun 19;2:e427. doi: 10.7717/peerj.427 (PMC4081156; doi:10.7717/peerj.427)
Supplement: Table S5 — Phenotypic variability amongst representative strains of P. sanctipauli sp. nov. +, positive; −, negative; w, weak [file peerj-02-427-s005.pdf]

Table S5. Phenotypic variability amongst representative strains of *P. sanctipauli* sp. nov. +, positive; -, negative; w, weak

Test of	A-373	A-379	A-394^{T}	A-397	A-398
Esterase (C 4)	-	+	-	+	+
Esterase lipase (C8)	+	+	-	+	+
Tryptophane deaminase	-	w	w	+	+
Indole production	-	w	-	+	+
Fermentation/oxidation:					
Citrate	-	-	-	w	w
D-Maltose	+	w	-	w	w
D-trehalose	+	w	-	+	+
D-Cellobiose	+	w	w	-	-
Sucrose	w	w	-	w	-
D-Melibiose	w	-	-	-	-
β-Methyl-D-Glucoside	-	-	-	w	w
D-Fructose	+	w	-	+	+
L-Rhamnose	w	-	-	-	-
D-Mannitol	+	-	-	-	-
Myo-Inositol	w	-	-	-	-
DNA G+C content (mol%)	47.2	48.4	48.2	49.4	49.7
