# Supplementary figures and images for: Photobacterium sanctipauli sp. nov. isolated from bleached Madracis decactis (Scleractinia) in the St Peter & St Paul Archipelago, Mid-Atlantic Ridge, Brazil
Source: PeerJ. 2014 Jun 19;2:e427. doi: 10.7717/peerj.427 (PMC4081156; doi:10.7717/peerj.427)

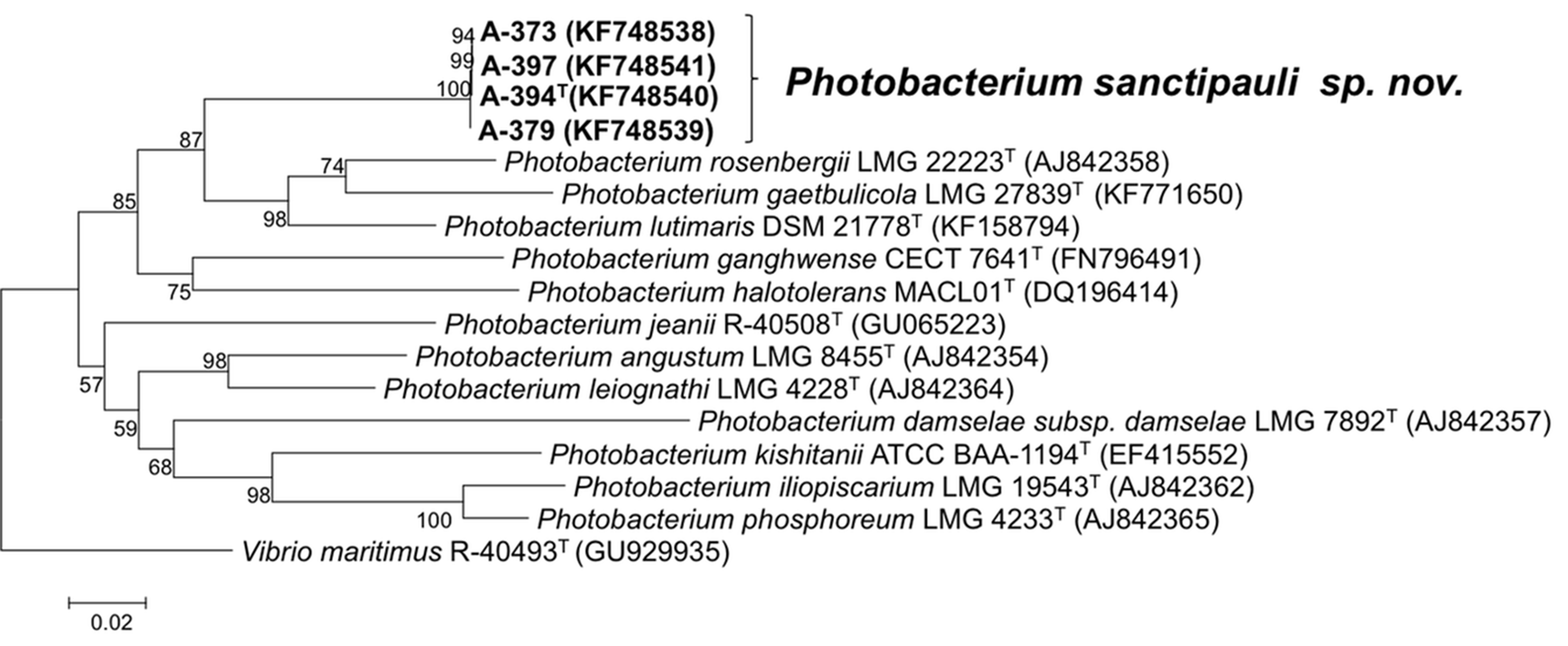

Supplement: Figure S1 — Neighbour-joining phylogenetic tree showing the position of P. sanctipauli sp. nov based on recA gene sequences (855 bp). The evolutionary distances were computed using the Kimura 2-parameter method. Phylogenetic analyses were conducted in MEGA5. Bootstrap values (>50%) shown are based on 1,000 repetitions. Vibrio maritimus R-40493T was used as outgroup. Bar, 2% estimated sequence divergence. [file peerj-02-427-s006.png]

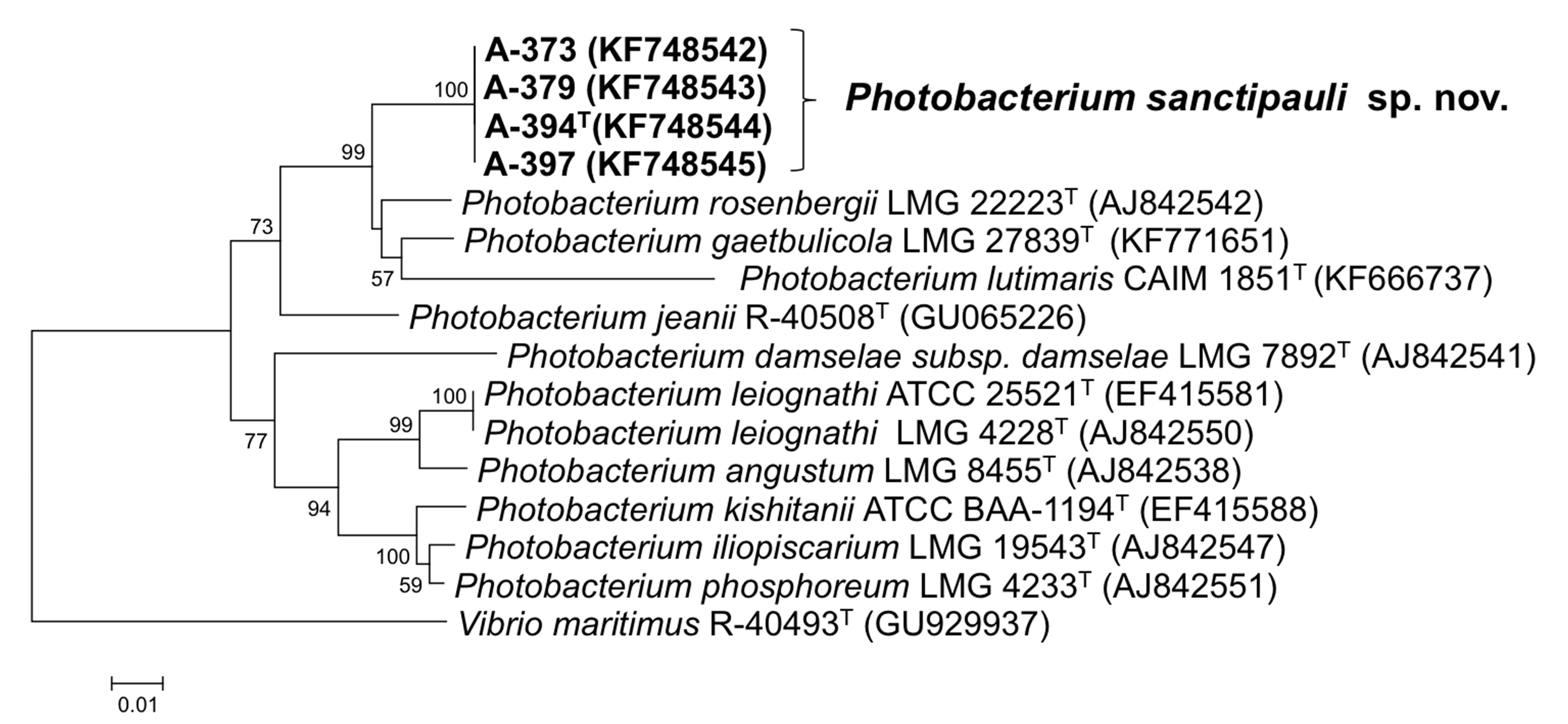

Supplement: Figure S2 — Neighbour-joining phylogenetic tree showing the position of P. sanctipauli sp. nov based on rpoA gene sequences (969 bp). The evolutionary distances were computed using the Kimura 2-parameter method. Phylogenetic analyses were conducted in MEGA5. Bootstrap values (>50%) shown are based on 1,000 repetitions. Vibrio maritimus R-40493T was used as outgroup. Bar, 1% estimated sequence divergence. [file peerj-02-427-s007.png]

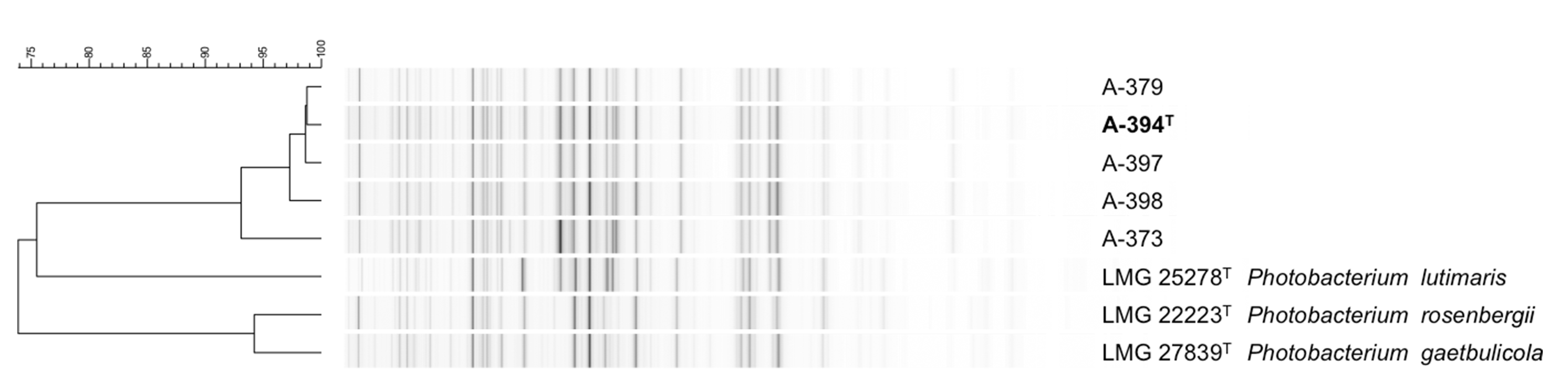

Supplement: Figure S3 — Comparison of the MALDI-TOF MS fingerprint profiles of the 5 novel strains (A−394T, A-373, A-379, A-397 and A-398) showing they are not clonal. The closely related type strains of P. rosenbergii (LMG 22223T), P. gaetbulicola (LMG 27839T) and P. lutimaris (LMG 25278T) were included in the analysis. The dendrogram was constructed using Pearson correlation coefficient and UPGMA. [file peerj-02-427-s008.png]
